# Ecocardiografia Transtorácica na Hipertensão Pulmonar: Ferramenta Fácil, mas é Preciso Cautela!

**DOI:** 10.36660/abc.20230380

**Published:** 2023-07-19

**Authors:** Hugo Hyung Bok Yoo

**Affiliations:** 1 Universidade Estadual Paulista Júlio de Mesquita Filho Faculdade de Medicina Botucatu São Paulo Brasil Universidade Estadual Paulista Júlio de Mesquita Filho – Faculdade de Medicina – Campus de Botucatu, Botucatu, São Paulo – Brasil

**Keywords:** Ecocardiografia, Hipertensão Pulmonar, Diagnóstico. Keywords: Echocardiography, Hypertension, Pulmonary, Diagnosis

A triagem de indivíduos de alto risco para hipertensão pulmonar (HP) geralmente começa com a ecocardiografia transtorácica (ETT) como o teste de primeira escolha para avaliação não invasiva do estado hemodinâmico vascular pulmonar. Portanto, a ecocardiografia pode fornecer informações abrangentes sobre a morfologia do coração, a função ventricular e anormalidades valvares.^1^

A ecocardiografia também é uma ferramenta útil para estimar a pressão arterial pulmonar sistólica (PSAP), as pressões do ventrículo direito (VD) e do átrio direito (AD) para atribuir um parâmetro ecocardiográfico da probabilidade de HP.^2^ Na maioria das vezes, a velocidade do jato de regurgitação tricúspide (VRT) é medida por ETT e, juntamente com uma estimativa da pressão atrial direita (PAD) baseada no colapso inspiratório e no tamanho da veia cava inferior, o jato VRT é usado para estimar a pressão sistólica da artéria pulmonar (PSAP).

No entanto, o detalhamento abrangente do mecanismo hemodinâmico subjacente da HP nem sempre é possível com a ecocardiografia e requer o cateterismo cardíaco direito (CCD).^2^

O diagnóstico definitivo de HP é feito pela medição invasiva das pressões pulmonares por CCD com mortalidade intra-hospitalar tão baixa quanto 0,055%.^3^ No entanto, a CCD em todos os pacientes sintomáticos seria impraticável e associada a custos elevados. Uma estimativa de HP não invasiva por ETT geraria um compromisso em relação a custo e simplicidade na prática diária.^4^

Afinal, qual a correlação entre a estimativa não invasiva (ETT) e invasiva (CCD) da PSAP e a determinação da necessidade de realização de CCD para avaliação diagnóstica de HP? Apesar da dúvida, poucos estudos com tamanho amostral pequeno investigam a correlação da PSAP com o CCD e questionam a precisão das estimativas ecocardiográficas.

Yock e Popp^5^ mostraram os primeiros dados com alta correlação (r = 0,93) entre a estimativa não invasiva e invasiva da PSAP em 54 pacientes. Em um estudo de coorte com 1.695 pacientes, Greiner et al.^6^ mostraram uma boa correlação para PSAP (r = 0,87) e uma precisão de 84,8%. Em uma população de 667 pacientes com HP ou doença pulmonar avançada, Amsallem et al.^7^ mostraram uma boa correlação de r = 0,84 com uma precisão de 72%. Em uma meta-análise, Taleb et al.^8^ analisaram nove estudos de pacientes (n = 20 a n = 150) com diferentes doenças (DPOC, fibrose pulmonar intersticial, apneia obstrutiva do sono, doença da válvula aórtica ou mitral, comunicação interatrial, etc.). As correlações entre a estimativa invasiva e não invasiva variaram de r = 0,65 a r = 0,97, com 40% e 78,5% de precisão.

Em uma grande coorte de 1.400 pacientes com estenose aórtica grave, a HP (pressão média da artéria pulmonar ≥25 mmHg) foi consistentemente diagnosticada considerando PSAP>40 mmHg por ecocardiografia. A sensibilidade para o diagnóstico de HP foi de 82,2%, com especificidade de 80,2%.^4^

Nesta edição, os autores,^9^ em um primeiro estudo de coorte retrospectivo brasileiro, compararam as pressões ETT e CCD em 95 pacientes com suspeita diagnóstica de hipertensão arterial pulmonar (HAP) e hipertensão pulmonar tromboembólica crônica (HPTEC) em contexto da prática clínica diária. Diferentes examinadores realizaram ETT e CCD com intervalo médio de 3,3 meses, mas todos os procedimentos foram realizados na mesma unidade cardiológica do hospital público de saúde.

Para descrever a concordância entre duas medidas quantitativas, os autores adotaram o método estatístico Bland-Altman.^10^ Eles mostraram um alto poder discriminatório de PSAP e VRT para diagnosticar HP. De acordo com suas definições (diferença de 10mmHg para PSAP e 5mmHg para PAD), apenas 33,4% das estimativas de PSAP e 55,1% das estimativas de PAD foram precisas. Esses achados semelhantes também foram demonstrados por Fisher et al.^11^ (PSAP: 52%), Rich et al.^12^ (PSAP: 49,4%) e REVEAL Registry^13^ (PSAP: 42,6% e RAP: 63,5%). Outros estudos mostraram medições ligeiramente mais confiáveis de PSAP e PAD (68% e 62%, respectivamente) em 79% dos pacientes.^14 , 15^

A PSAP depende da função sistólica do ventrículo direito (VD) e do volume sistólico. Em estágios avançados de HP, a função do VD se deteriora, o que pode diminuir o grau de elevação da PSAP e levar a uma subestimação da resistência vascular pulmonar (RVP). Portanto, embora o ETT seja o teste de triagem padrão para HP, ele não é totalmente preciso e ainda há incerteza sobre quais medidas ecocardiográficas são mais confiáveis e úteis na prática diária.

Alguns fatores, como população heterogênea, diferentes intervalos de tempo realizados entre ETT e CCD e profissionais com diferentes experiências, podem explicar em parte as discrepâncias nas correlações e acurácias relatadas entre os estudos. As modalidades de ecocardiografia aumentaram significativamente, fornecendo informações importantes sobre a estrutura e função do coração direito em pacientes com HP. No entanto, esta ferramenta não foi definitivamente validada como um substituto completo para CCD em pacientes com HP.

A falta de correlação entre os parâmetros seriados de ETT e CCD reforça a importância de não se basear em um único teste na avaliação de pacientes com HP. Além disso, é necessário cautela para o uso fácil e acessível do ETT, pois sempre permanece uma alternativa potencialmente mais atraente do que técnicas diagnósticas mais complicadas, invasivas ou caras.
