# Supplementary material for: Ecocardiografia Transtorácica na Hipertensão Pulmonar: Ferramenta Fácil, mas é Preciso Cautela!
Source: Arq Bras Cardiol. 2023 Jul 19;120(7):e20230380. [Article in Portuguese] doi: 10.36660/abc.20230380 (PMC10382149; doi:10.36660/abc.20230380)
Supplement: Translation

# Translation: Transthoracic Echocardiography in Pulmonary Hypertension: Easy Tool, but Caution is Needed!

**Authors:** Hugo Hyung Bok Yoo

**Affiliations:** 1 Brasil Universidade Estadual Paulista Júlio de Mesquita Filho – Faculdade de Medicina – Campus de Botucatu, Botucatu, São Paulo – Brasil

**Keywords:** Transthoracic, echocardiography, pulmonary, hypertension

Screening high-risk individuals for pulmonary hypertension (PH) usually begins with transthoracic echocardiography (TTE) as the first-choice test for noninvasive pulmonary vascular hemodynamic status evaluation. Therefore, echocardiography can provide comprehensive information on heart morphology, ventricular function, and valve abnormalities.^1^

Echocardiography is also a useful tool for estimating the systolic pulmonary arterial pressure (sPAP), right ventricular (RV), and right atrial (RA) pressures to assign an echocardiographic parameter of the probability of PH.^2^ Most often, the tricuspid regurgitation jet velocity (TRJV) is measured by TTE and, together with an estimate of the right atrial pressure (RAP) based on the inspiratory collapse and the size of the inferior vena cava, the TRJV jet is used to estimate the systolic pulmonary artery pressure (sPAP).

Nevertheless, comprehensive detail of the underlying hemodynamic mechanism of PH is not always possible with echocardiography and requires the right heart catheterization (RHC).^2^

Definitive diagnosis of PH is made by invasive measurement of pulmonary pressures by RHC with in-hospital mortality as low as 0.055%.^3^ However, RHC in all symptomatic patients would be impractical and associated with high costs. A noninvasive HP estimation by TTE would provide a compromise regarding cost and simplicity in daily practice.^4^

After all, what is the correlation between noninvasive (TTE) and invasive (RHC) estimation of sPAP and the determination of the need to perform RHC for the diagnostic evaluation of PH? Despite the doubt, few studies with small sample sizes investigate the correlation of sPAP with RHC and question the accuracy of echocardiographic estimates.

Yock and Popp^5^ showed the first data with a high correlation (r = 0.93) between the noninvasive and invasive estimation of sPAP in 54 patients. In a cohort study with 1,695 patients, Greiner et al.^6^ showed a good correlation for sPAP (r = 0.87) and an accuracy of 84.8%. In a population of 667 patients with PH or advanced lung disease, Amsallem et al.^7^ showed a good correlation of r = 0.84 with an accuracy of 72%. In a meta-analysis, Taleb et al.^8^ analyzed nine studies of patients (n = 20 to n = 150) with different diseases (COPD, interstitial pulmonary fibrosis, obstructive sleep apnea, aortic or mitral valve disease, atrial septal defect, etc.). Correlations between invasive and noninvasive estimation ranged from r = 0.65 to r = 0.97, with 40% and 78.5% accuracy.

In a large cohort of 1400 patients with severe aortic stenosis, PH (a mean pulmonary artery pressure ≥25mmHg) was consistently diagnosed considering sPAP>40 mmHg by echocardiography. The sensitivity for diagnosing PH was 82.2%, with a specificity of 80.2%.^4^

In this issue, the authors,^9^ in a first Brazilian retrospective cohort study, compared TTE and RHC pressures in 95 patients with a suspected diagnosis of pulmonary arterial hypertension (PAH) and chronic thromboembolic pulmonary hypertension (CTEPH) in the context of daily clinical practice. Different examiners performed TTE and RHC at an average interval of 3.3 months, but all procedures were performed in the same cardiac unit of the public health hospital.

To describe the agreement between two quantitative measures, the authors adopted the Bland-Altman statistical method.^10^ They showed a high discriminatory power of sPAP and VRT for diagnosing PH. According to their definitions (10mmHg difference for sPAP and 5mmHg for RAP), only 33.4% of the sPAP and 55.1% of the RAP estimates were accurate. These similar findings were also demonstrated by Fisher et al.^11^ (sPAP: 52%), Rich et al.^12^ (sPAP: 49.4%), and REVEAL Registry^13^ (sPAP: 42.6% and RAP: 63.5%). Other studies have shown slightly more reliable measurements of sPAP and RAP (68% and 62%, respectively) in 79% of patients.^14 , 15^

The sPAP depends on right ventricular (RV) systolic function and stroke volume. In advanced stages of PH, RV function deteriorates, which may decrease the degree of sPAP elevation and lead to an underestimation of pulmonary vascular resistance (PVR). Therefore, although TTE is the standard screening test for PH, it is not completely accurate, and there is still uncertainty about which echocardiographic measurements are most reliable and useful in daily practice.

Some factors, such as heterogeneous population, different time intervals performed between TTE and RHC, and professionals with different experiences, may partly explain the discrepancies in correlations and accuracies reported between studies. Echocardiography modalities have increased significantly, providing important information about the structure and function of the right heart in patients with PH. However, this tool has not been definitively validated as a complete replacement for RHC in patients with PH.

The lack of correlation between serial TTE and RHC parameters reinforces the importance of not relying on a single test when evaluating patients with PH. In addition, caution is needed for the easy and accessible use of the TTE, as it always remains a potentially more attractive alternative than more complicated, invasive, or expensive diagnostic techniques.
